# Comparison of subcutaneous and intravenous administration of the monoclonal antibody CSX-1004 on fentanyl-induced respiratory depression in squirrel monkeys

**DOI:** 10.3389/fphar.2026.1964162

**Published:** 2026-10-30

**Authors:** Mukesh Kumar, Emily L. Burke, Yanan Zang, Abhisheak Sharma, Paul T. Bremer, Rajeev I. Desai

**Affiliations:** 1 Department of Psychiatry, Harvard Medical School, Boston, MA, United States; 2 Integrative Neurochemistry Laboratory, Behavioral Biology Program, McLean Hospital, Belmont, MA, United States; 3 Department of Pharmaceutics, College of Pharmacy, University of Florida, Gainesville, FL, United States; 4 Cessation Therapeutics, Inc, San Diego, CA, United States

**Keywords:** CSX-1004, fentanyl, monoclonal antibody, opioid overdose, Pharmacodynamics, pharmacokinetics, population modeling, respiratory depression

## Abstract

**Background:**

Fentanyl and related synthetic opioids remain major contributors to overdose mortality, primarily through opioid-induced respiratory depression. CSX-1004 is a fully human monoclonal antibody designed to bind fentanyl in the peripheral circulation and prevent its entry into the brain. This study evaluated the pharmacokinetic (PK) and pharmacodynamic (PD) equivalence of intravenous (IV) and subcutaneous (SC) administration of CSX-1004 in squirrel monkeys and established a population PK (PopPK) model to support route extrapolation.

**Methods:**

Protection against fentanyl-induced respiratory depression was assessed after administration of 40 mg/kg CSX-1004 using whole-body plethysmography over 28 days. Serum antibody concentrations were quantified by enzyme-linked immunosorbent assay, and PK parameters were characterized using noncompartmental and PopPK analyses.

**Results:**

Both IV and SC administration produced substantial and durable attenuation of fentanyl-induced respiratory depression, resulting in a greater than 10-fold rightward shift in fentanyl potency that persisted for up to 28 days. SC CSX-1004 administration exhibited a maximum concentration of 491.7 ± 50.4 μg/mL at 48.0 ± 19.6 h (T_max_) and an absolute bioavailability of 92.0% based on ${AUC}_{0-\infty }$ . Terminal half-life was comparable between routes (∼272 h). A two-compartment PopPK model with first-order absorption and elimination adequately described the concentration–time data and demonstrated robust predictive performance. Serum concentrations and respiratory protection were lower after SC than after IV administration during the first 24–48 h, reflecting the absorption phase, and converged from Day 2 onward. Simulations indicated that SC and IV administration achieved overlapping systemic exposures beyond the absorption phase and equivalent concentrations associated with sustained respiratory protection through Day 28.

**Conclusion:**

These findings demonstrate that SC administration of CSX-1004 provides PK and PD outcomes comparable to IV dosing, once absorption is complete, supporting SC delivery as a practical and clinically feasible approach for long-acting prophylaxis against fentanyl-induced respiratory depression.

## 1 Introduction

Opioid overdoses are a major public health crisis in the United States and across the world, with synthetic opioids like fentanyl and fentanyl analogs contributing disproportionately to the rising number of deaths in the United States and around the world (Ahmad et al., 2023). Fentanyl is highly potent, as it has high affinity at the mu-opioid receptor (MOR) and therefore activates MORs, causing feelings of euphoria, as well as potential adverse effects like respiratory depression (Comer and Cahill, 2019). Fentanyl’s high potency, coupled with lipophilicity, allows it to quickly act on MORs, making overdose and mortalities more common than other opioids opioids (Gill et al., 2019). The gold standard treatment for opioid overdoses is naloxone, a MOR antagonist (Bird et al., 2023; Comer and Cahill, 2019). Although naloxone has been shown to successfully reverse the effects of fentanyl in most cases, additional naloxone doses are sometimes needed to reverse a severe opioid overdose caused by ultrapotent synthetic opioids (Dahan et al., 2024; Rzasa Lynn and Galinkin, 2018). Moreover, naloxone has a shorter duration of action compared to some fentanyl analogs, posing an additional challenge for treating overdoses from longer acting opioids such as carfentanil (Moss and Carlo, 2019). Such issues illustrate the duration-of-action limitations that constrain short-acting antagonist therapy.

To address these limitations of naloxone for overdose reversal, we have investigated alternative strategies for preventing fentanyl overdose. One such approach is the use of monoclonal antibodies (mAbs), which can specifically bind fentanyl and fentanyl analogs in the periphery, blocking the drug from crossing the blood brain barrier (Smith et al., 2019; Baehr et al., 2020; Ban et al., 2021; Hicks et al., 2022; Baehr et al., 2022; Chen et al., 2024; Baehr et al., 2025; Galbo-Thomma et al., 2025). CSX-1004, a fully human mAb, has been shown to successfully block fentanyl’s respiratory, behaviorally disruptive, and antinociceptive effects (Bremer et al., 2023). While these initial studies focused on intravenous (IV) administration of CSX-1004, this route of administration is not easily deployable in most emergency and outpatient settings, and therefore, other alternative routes of administration are necessary to explore. Subcutaneous (SC) administration would potentially offer a more accessible way to administer the mAb, expanding its potential use as a long-acting prophylactic administered in outpatient, community, or opioid use disorder (OUD) treatment settings to individuals at high risk of repeated fentanyl exposure. Because absorption of subcutaneously administered IgG is slow relative to the time course of an acute overdose, the SC route appears better suited to pre-exposure prophylaxis than to acute overdose reversal, for which naloxone remains the standard of care; thus, its intended role is to provide durable protection during the interval following a nonfatal overdose in which the risk of recurrent opioid exposure is greatest (Weiner et al., 2020). Beyond improved accessibility, the SC route is associated with enhanced patient compliance and reduced healthcare costs (George et al., 2025). However, the absorption of biotherapeutics following SC injection is often slower and governed by different physiological constraints than the immediate systemic availability provided by an IV bolus injection (Bittner et al., 2018; Davis et al., 2024; Varkhede and Forrest, 2018). Consequently, a rigorous quantitative framework is required to bridge these administration routes and ensure therapeutic efficacy is maintained. To address these complexities, model-informed pharmacokinetic (PK) bridging has emerged as an established strategy for evaluating alternative administration routes (Hourcade-Potelleret et al., 2014). By integrating concentration-time profiles with a population-based approach, population PK (PopPK) modeling facilitates the characterization of inter-individual and residual unexplained variabilities, and the identification of factors that govern mAb absorption, disposition, and overall bioavailability (van der Zeeuw et al., 2026).

Although the efficacy of CSX-1004 after IV administration has been established, it is not known whether SC administration delivers systemic exposure sufficient to reproduce that efficacy, how rapidly protection is established relative to the IV route, or what absolute bioavailability is achieved; nor has a quantitative framework been available to bridge the two routes or to extrapolate exposure to untested dose levels. The present study was designed to address this gap. The current study aimed to evaluate whether the pharmacokinetics (PK) and efficacy of CSX-1004 are comparable following IV and SC administration at a dose of 40 mg/kg in squirrel monkeys. In addition, model-based simulations were performed to predict PK outcomes across a range of antibody doses (10, 40, and 80 mg/kg). Experimentally, whole-body plethysmography was used as a translationally relevant pharmacodynamic (PD) readout to examine CSX-1004 blockade of fentanyl-induced respiratory depression in squirrel monkeys over a 28-day period after IV and SC administration of 40 mg/kg. To characterize the observed variability in disposition and quantify the influence of administration route, a PopPK model was developed. This model was subsequently utilized to simulate PK profiles across a broader range (10, 40, and 80 mg/kg) for both routes. These simulations focused on predicting the mAb concentrations at 672 h (C_672_) following IV administration at 40 mg/kg, serving as a C_trough_ surrogate, to evaluate the probability of maintaining therapeutic concentrations after SC administration required for sustained protection from fentanyl-induced respiratory depression throughout the 28-day study interval.

## 2 Methods

### 2.1 Animals

All research complies with all ethical regulations and all animal study protocols, which were approved before the study. Eight adult squirrel monkeys (*Saimiri sciureus*; five males and three females, 8–18 years of age, 0.65–1.0 kg) were used as subjects in the whole-body ventilation study (Bremer et al., 2023). Individual treatment conditions were studied in subgroups (n = 3/4) of animals, and some animals contributed to more than one treatment condition (i.e., both 40 mg/kg IV and SC treatment), separated by the washout intervals described in Section 2.3.2. All monkeys were individually housed in stainless steel cages in a climate-controlled vivarium (lights on at 7 a.m. to 7 p.m.) in the McLean Hospital Animal Care Facility. The facility is licensed by the U.S. Department of Agriculture and compliant with guidelines provided by the Committee on Care and Use of Laboratory Animals of the Institute of Laboratory Animal Resources, Commission on Life Sciences (National Research Council (US) Committee for the Update of the Guide for the Care and Use of Laboratory Animals, 2011). Except during testing, subjects had unrestricted access to water in the home cage and were also fed a daily allotment of high-protein primate chow (Purina Monkey Chow, St. Louis, MO), supplemented with fruit and multivitamins in the home cage. Daily allotments of food were adjusted to maintain recommended body weights. All experiments were conducted between the hours of 8 a.m. to 6 p.m. under protocols approved by the Institutional Animal Care and Use Committee (IACUC) at McLean Hospital. All subjects had previously participated in operant studies and had received acute injections of drugs of different pharmacological classes (e.g., psychomotor stimulants) but were not active in any studies for at least 3 months before this study.

### 2.2 Material

Fentanyl hydrochloride was provided from the NIDA/NIH Drug Supply Program (Division of Therapeutics and Medical Consequences, NIDA Resources Drug Supply Program). CSX-1004 was manufactured by KBI Biopharma (Durham, NC) on behalf of Cessation Therapeutics and provided as a nominal 100 mg/mL solution in isotonic buffer (Lot 210618–0040-BDS). Testing results of this lot met or exceeded minimum acceptance criteria for appearance (clear, slightly yellow, free of visible particles), pH, purity (SEC HPLC main peak: 98.8%, reduced CE-SDS: 97.89%, potency (100% fentanyl-BSA binding vs. reference material) and strength. Residual host cell protein, DNA, protein A, along with endotoxin were quantified and found to be below acceptable limits. CSX-1004 was thawed as needed from −80 °C storage and diluted in sterile saline immediately before administration; the required volume was calculated from the nominal concentration and the individual body weight. Fentanyl solutions were freshly prepared on a weekly basis and cumulative doses of fentanyl were administered intramuscularly (IM) based on body weight. Doses of all compounds are expressed in terms of the free base.

### 2.3 Whole-body plethysmography procedure

#### 2.3.1 Apparatus

Ventilation studies were conducted as described previously (Bremer et al., 2023; Wang et al., 2020) in a custom-made acrylic chamber (9.5″d x 10″w x 10″h) that served as a whole-body plethysmograph (EMKA Technologies, Montreal, PQ, Canada). A continuous flow of 5 L/min of either air or air mixed with 5% CO_2_ was introduced to the chamber through a port at the front of the chamber and removed at the same rate through a similar port in the rear of the chamber. Changes in flow inside the plethysmograph were recorded by a pressure transducer connected to a computer; SCIREQ IOX2 software converted pressure displacement to measures of breathing frequency, tidal volume, and minute volume.

#### 2.3.2 Ventilation procedure

This experiment was designed to establish, within each subject, the fentanyl dose-effect function for respiratory depression before antibody treatment, so that the magnitude and duration of CSX-1004 blockade could subsequently be quantified as a rightward and downward shift of that same function in the same animals. Prior to mAb administration, baseline ventilatory responses induced by fentanyl were established. To generate fentanyl dose-response functions in the respiratory depression assay, experimental sessions consisted of five successive test cycles. An IM saline injection was given immediately prior to the first cycle, which included respiration measurements in air (20 min) followed by 5% CO_2_ in air (10 min). The extended period within the air condition during the first cycle allowed for habituation to the chamber and conditions. Next, up to four IM cumulative doses of fentanyl were given, each followed by measurement in the plethysmography chamber in air (10 min) and then a mixture of 5% CO_2_ and air, and respiratory rate and tidal volume (ml/breath) were recorded over 1 min periods and multiplied to obtain minute volumes. Each exposure to 5% CO_2_ in air lasted 10 min, and each cumulative fentanyl injection was administered immediately following the preceding CO_2_ exposure. The first (habituation) cycle therefore lasted 30 min and each of the four subsequent cycles lasted 20 min (10 min in air followed by 10 min in 5% CO_2_ in air), so that successive cumulative fentanyl doses were separated by approximately 20 min and a complete dose-effect session lasted approximately 110 min. Ventilatory measures used for analysis were taken from the final 3 min of each air and each 5% CO_2_ exposure. This timing is identical to that used previously in this species and apparatus (Bremer et al., 2023).

Following this, subjects were administered CSX-1004 either IV or SC, and the attenuation of the fentanyl respiratory response by pre-treatment with CSX-1004 was determined. The animals were then retested using the above-described respiration procedure with one saline injection followed by four injections of cumulative doses of fentanyl (0.001–0.018 mg/kg). The identical fentanyl challenge testing procedure occurred six times over the course of a 28-day period, starting at 30 min post-mAb administration along with Days 2, 7, 14, 21, and 28 to determine the duration of both IV and SC CSX-1004’s action. To account for small variations in normal respiration over time, minute volumes were normalized as a percent to the within-subject saline response minute volume gathered on each given test day. In addition, blood sampling for PK analysis was conducted on Day 0 at 5 min, 3, 6, and 24 h, post-administration, with subsequent samples collected on each of the same PD testing days through Day 28. On Days 2–28, blood was drawn immediately before the start of the fentanyl dose-effect determination, so that the measured serum CSX-1004 concentration reflects antibody exposure at the time of the fentanyl challenge rather than after it; for Day 0, the 6 h sample was used to characterize exposure during the initial challenge session. Baseline fentanyl dose-effect functions were re-established after each antibody treatment using a washout interval of at least 62 days (range 62–128 days), which has previously been shown to be sufficient for complete reversal of CSX-1004 effects on the fentanyl dose-effect function in this species (Bremer et al., 2023).

### 2.4 ELISA method for quantifying CSX-1004 in non-human primates (NHPs)

Corning 3690 half-area, 96-well microtiter plates were coated with 12.5 ng fentanyl-BSA antigen in water per well and then dried overnight at 37 °C. After blocking in 3% BSA in PBS-T, six 1:2 serial dilutions of each serum sample starting at 1:1200 were run alongside six 1:2 dilutions of CSX-1004 antibody in duplicate starting at 100 ng/mL as a standard curve. Serum samples and CSX-1004 antibody were both diluted in 1% BSA in PBS-T as was the secondary antibody. After a 1 h incubation at 37 °C, plates were washed 5 times with PBS-T, and 1:10,000 goat anti-human IgG HRP (1 mg/mL, Southern Biotech #204005, Birmingham, AL) was added and incubated for 0.5 h at 37 °C. After washing 5 times with PBS-T, TMB substrate (Thermo Fisher #34021, Waltham, MA) was added and plates were stopped using 2 M sulfuric acid and then read at 450 nm using a SpectraMax i3x plate reader running SoftMax Pro version 7.0 software.

### 2.5 Non-compartmental analysis (NCA)

NCA was performed using Phoenix 8.3 (Certara, Princeton, NJ) to characterize the PK profile of CSX-1004 following 40 mg/kg IV and 40 mg/kg SC administration. While the maximum serum concentration (C_max_ or C_0_) and time to reach maximum serum concentration (T_max_) were extracted directly from the observed serum concentration-time data, secondary PK parameters, including the area under the concentration-time curve (AUC_0−last_ and ${AUC}_{0-\infty }$ ), clearance (CL), volume of distribution (V_d_), and elimination half-life (t_1/2_) were derived based on the standard NCA procedures.

### 2.6 Absolute bioavailability

The absolute bioavailability is the dose-normalized area under the curve ( ${AUC}_{0-\infty }$ ) of the extravascular route divided by the ${AUC}_{0-\infty }$ of the IV route (Kumar et al., 2023). The formula for calculating the % absolute bioavailability (F) of a drug administered SC is given below as Equation 1.

$$F=\frac{{\text{AUC}}_{0-\infty \text{SC}}\;*\;{\text{Dose}}_{\text{IV}}}{{\text{AUC}}_{0-\infty \text{IV}}\;*\;{\text{Dose}}_{\text{SC}}}\;*\;100$$ (1)

### 2.7 Population PK (PopPK) analysis

#### 2.7.1 Data sources and software

PK data for IV administration were obtained from prior NHP studies (Bremer et al., 2023; Yassen et al., 2007), SC administration data were generated from the current investigation. PopPK analysis was conducted using a nonlinear mixed-effects modeling approach in Monolix Suite 2024 R1 (Lixoft, Antony, France). Parameter estimation, including typical population values, between-subject variability (BSV), and residual unexplained variability (RUV), was performed using the Stochastic Approximation Expectation-Maximization (SAEM) algorithm (Delyon et al., 1999). Data exploration, graphical visualization, and simulations were executed in R (version 4.6.0) using RStudio (version 2026.04.0).

#### 2.7.2 Model development

A population PK model was developed by simultaneously analyzing CSX-1004 concentration–time data following IV and SC administration. Model development comprised establishment of the base model, including disposition, SC absorption, and variability components, followed by covariate evaluation. One- and two-compartment disposition models with linear and non-linear (Michaelis-Menten) elimination were evaluated iteratively by comparing diagnostic plots and objective function values (OFV; −2 log-likelihood). SC absorption was described by a first-order process characterized by an absorption rate constant (K_a_) and absolute bioavailability (F). F was estimated on the logit scale, constraining it to the interval (0, 1), with no inter-individual variability estimated for F. Inter-individual variability (IIV) in PK parameters was described using an exponential model, assuming a log-normal distribution of individual parameter values (Equation 2):

$$\theta_{i}=\theta_{pop}\cdot e^{\eta_{i}}$$ (2)

Where *θ*_*i*_ represents the parameter for the *i*th individual, *θ*_*pop*_ is the typical population parameter value, and *η*_i_ is the individual random effect and is assumed to be normally distributed with a mean of 0 and a variance of ω^2^ (Zhu et al., 2026). Residual unexplained variability (RUV) was assessed using additive, proportional, and combined error structures to determine the optimal residual error model (Mould and Upton, 2013). Once the base model was established, body weight and treatment group (10 mg/kg IV, 40 mg/kg IV, and 40 mg/kg SC) were evaluated as potential covariates using manual stepwise forward inclusion followed by backward elimination. Forward inclusion of a covariate required a statistically significant drop in the OFV (p < 0.05, ΔOFV >3.84 for df = 1; ΔOFV >5.99 for df = 2) alongside improved goodness-of-fit (GOF) diagnostics and reduced inter-individual variance (ω) (Chasseloup et al., 2020). Model parsimony was confirmed via backward elimination, retaining a covariate only if its removal caused significant OFV inflation or clear structural degradation of the diagnostic profiles.

#### 2.7.3 Model evaluation

Model selection and robustness were assessed using the precision of parameter estimates (Relative Standard Errors, RSE%), minimization of the objective function value (OFV) and BIC, and standard graphical diagnostics. Goodness-of-fit plots included observed versus predicted concentrations, residual plots, and visual predictive checks (VPCs). The accuracy and robustness of the estimated parameters were confirmed via bootstrapping with 1000 replications.

#### 2.7.4 Simulations

Stochastic population simulations (n = 500 virtual individuals per dose level) were conducted using the final PopPK model to predict the 28-day systemic exposure profiles of CSX-1004 across 10, 40, and 80 mg/kg dose levels. All population simulations and subsequent graphical visualizations were executed within R (version 4.6.0) utilizing the *mrgsolve* and *ggplot2* packages.

### 2.8 Data analysis and statistics

A within-subject design was used, in which each subject served as its own control and received all relevant test and control conditions. In respiration studies, data from the last 3 min of air or 5% CO_2_ mixtures were used for analysis of drug effects on ventilation. Minute volume (V_E_) was calculated by multiplying the frequency of ventilation (f) (respiration rate) by the tidal volume (V_T_) [V_E_ = V_T_ X f]. The V_E_ data were normalized to within-subject saline values and expressed as a percentage of the saline value (% Saline = V_E test_/V_E saline_ X 100). All data were examined at individual and group levels of analysis using GraphPad Prism 8. Data points on graphs are expressed as group means ± SEM. Normalized minute volume was analyzed by two-way repeated-measures ANOVA with treatment condition (pre-antibody, IV CSX-1004, SC CSX-1004) and fentanyl dose as within-subject factors, followed by Bonferroni’s multiple-comparisons test; planned comparisons were made between each CSX-1004 condition and the pre-antibody baseline, and between the IV and SC conditions, at every fentanyl dose on every test day. Serum CSX-1004 concentrations were compared between routes by two-way repeated-measures ANOVA (route × sampling time) with Bonferroni’s correction. Effects were considered significant at P < 0.05. Sex was evaluated as a between-subject factor in all analyses; no significant sex-related differences were detected in any NHP experiment, and data from male and female subjects were therefore pooled.

ELISA data were analyzed using GraphPad PRISM eight using a 4-PL fit of the CSX-1004 standards to determine unknown serum concentrations of CSX-1004. At least 2–3 dilutions per sample in the working range of the standard curve were used for interpolation and averaged to obtain the values as presented. PK analysis of mAb serum concentrations was performed in Phoenix WinNonlin version 8.3 to obtain the PK parameters.

## 3 Results

### 3.1 Both IV and SC CSX-1004 attenuate Fentanyl’s respiratory depressant effects

We evaluated the ability of CSX-1004 to block the respiratory depressant effects of fentanyl, using a whole-body plethysmography procedure. Test sessions were conducted under a normal air atmosphere as well as a mixture of 5% CO_2_ and air to increase ventilatory response and sensitivity to opioids. Results showed that both IV and SC routes of administration provided protection against fentanyl’s respiratory depressant effects in a time-dependent manner for up to 28 days after treatment (Figure 1). Both SC and IV administration resulted in a significant flattening of the fentanyl dose‐response curve compared to pre-antibody levels, indicating a >10-fold rightward shift in potency. The maximal blockade magnitude was within the first 7 days of IV and SC administration of 40 mg/kg CSX-1004. The two routes differed only during the initial absorption phase. On Day 0, attenuation at the highest fentanyl dose tested (0.018 mg/kg) was smaller after SC than after IV administration (approximately 72% versus 86% of the within-subject saline minute volume), consistent with the substantially lower serum antibody concentration present at that time after SC dosing (Figure 2). From Day 2 onward, once SC absorption was essentially complete, the dose-effect functions for the two routes were superimposable and no significant route difference was detected at any fentanyl dose. These data indicate that SC administration reproduces the efficacy of IV administration once the absorption phase is complete but does not provide fully equivalent protection within the first hours after injection.

**FIGURE 1:**
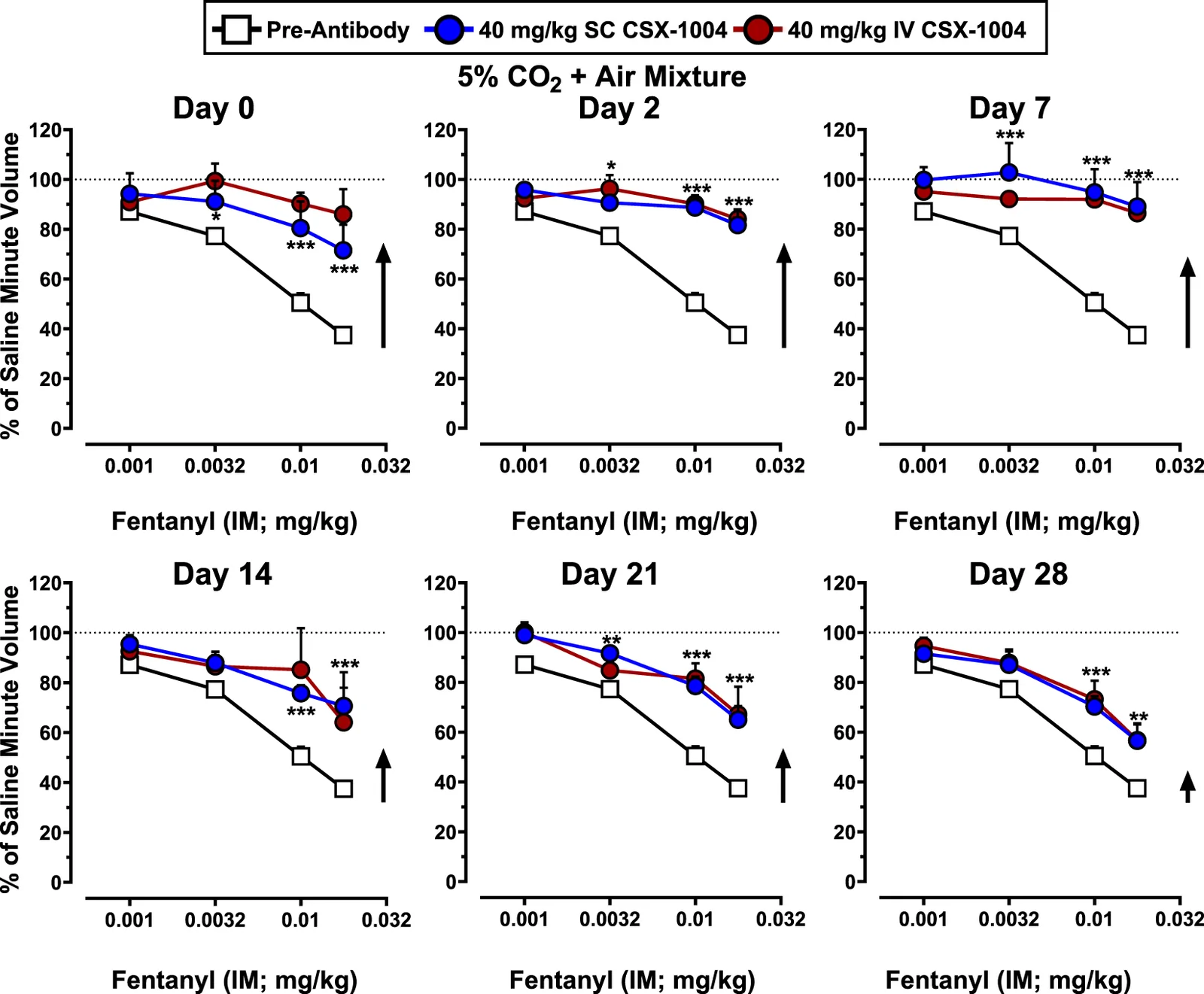
CSX-1004 provides durable protection from fentanyl respiratory depression in squirrel monkeys. Time-dependent effects of IV 40 mg/kg CSX-1004 (red circles) and SC 40 mg/kg CSX-1004 (blue circles) on fentanyl respiratory depression over 28 days compared to pre-antibody baseline effects of fentanyl (white squares). The values shown are expressed as a percentage of minute volume from a saline injection (100%, dotted line) obtained on each test day from n = 4 (IV) and n = 3 (SC) monkeys per treatment condition, drawn from the cohort of eight animals described in Section 2.1. Arrows are shown to approximate the magnitude of CSX-1004 efficacy at each time point. Statistics were determined by a two-way RM ANOVA [F (6, 12) = 6.877, P = 0.0024, effect over time], with Bonferroni’s post hoc test comparing respiration in SC CSX-1004-treated vs. pre-antibody at each fentanyl dose level (0.001, 0.0032, 0.01, 0.018 mg/kg) *P < 0.05, **P < 0.01, ***P < 0.001. No statistically significant differences were detected in comparing IV vs. SC data. Data points and error bars represent means ± SEM.

**FIGURE 2:**
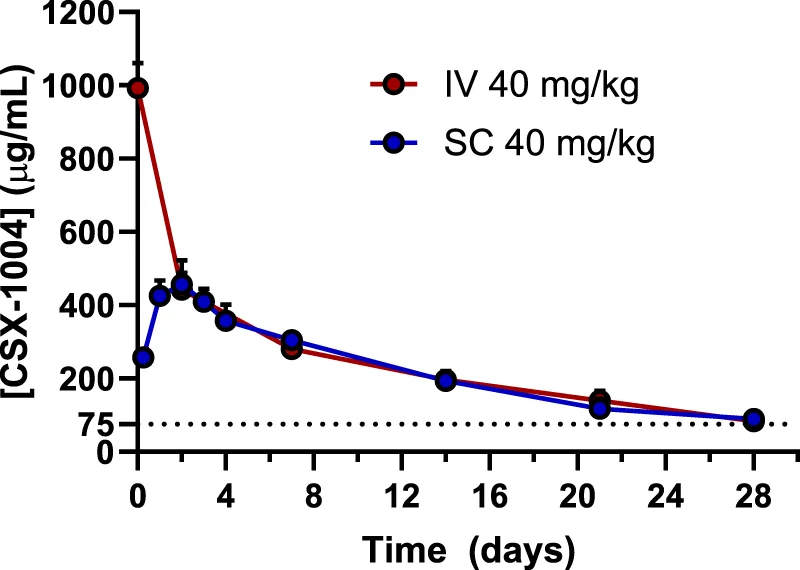
Serum concentration-time profiles of CSX-1004 after a 40 mg/kg dose administered by the SC and IV routes (mean ± SD). Error bars represent SD; where error bars are not visible the SD is smaller than the plotting symbol. The dotted line indicates 75 μg/mL. The AUCSC/AUCIV ratio calculated from AUC_0–672_ h is 0.87. The effect of injection route on plasma concentrations was not statistically different by a two-way RM ANOVA.

### 3.2 Serum concentrations of CSX-1004 following IV and SC administrations

A fully validated ELISA method was used to quantify serum concentrations of CSX-1004 over a 28-day following 40 mg/kg administration, and the PK profiles of both the IV and SC delivery of CSX-1004 are depicted in Figure 2. Serum concentrations during the first 24 h were markedly lower after SC than after IV administration, reflecting the absorption phase, and did not differ significantly between routes from Day 2 onward (two-way repeated-measures ANOVA, route × time). The interval over which the two concentration-time profiles converge corresponds to the interval over which the respiratory dose-effect functions converge (Figure 1), and the terminal phases of the two profiles are essentially superimposable.

### 3.3 Pharmacokinetic profile and absolute bioavailability

NCA was utilized to characterize the PK profiles of CSX-1004 across the evaluated routes (Table 1). Following IV administration, plasma concentrations peaked immediately, reflecting instantaneous systemic availability. Conversely, SC administration exhibited a protracted absorption phase, reaching a maximum plasma concentration (C_max_) of 491.7 ± 50.4 μg/mL at a T_max_ of 48.0 ± 19.6 h, post-dose.

**TABLE 1:** Pharmacokinetic parameters of CSX-1004 following IV or SC administration in squirrel monkeys.

Parameter	IV (40 mg/kg)	SC (40 mg/kg)
C_0_ or C_max_ (µg/mL)	1009.3 ± 102.6	491.7 ± 50.4
T_max_ (h)	-	48.0 ± 19.6
T_1/2_ (h)	272.1 ± 92.5	271.4 ± 76.3
AUC_0-672_ (h*µg/mL)	165,714.6 ± 19,700.7	144,672.3 ± 31,467.4
AUC_0-∞_ (h*µg/mL)	198,819.0 ± 24,894.8	182,993.7 ± 52,563.7
V_z_ (mL/kg)	78.6 ± 22.1	-
CL (mL/h/kg)	0.21 ± 0.05	-
F%	-	92.0

Data expressed as mean ± SD; - represents not applicable. C_0_ and C_max_ expressed as serum concentration for the first time-point after IV, dose and peak serum concentration after SC administration; CL, and V_z_ are expressed per kilogram of body weight, consistent with the values reported for this cohort in Bremer et al. (2023). F% represents absolute bioavailability, calculated as the dose-normalized ${AUC}_{0-\infty }$ of the SC, route divided by that of the IV route at 40 mg/kg; the extrapolated portion of ${AUC}_{0-\infty }$ was 16.6% (IV) and 20.9% (SC). Parameters are reported to a consistent number of decimal places within each row.

For the 40 mg/kg IV, the mean apparent volume of distribution (V_z_ = 78.6 mL/kg) and low systemic clearance (CL = 0.21 mL/h/kg) are highly characteristic of macromolecular disposition, indicating primary confinement to the vascular and extracellular fluid compartments with minimal tissue distribution. The apparent volume of distribution therefore approximates total blood volume, and the model-estimated central volume (V1 = 19.1 mL) approximates plasma volume, consistent with a molecule largely restricted to the vascular and interstitial space. These parameters sustained a prolonged, route-independent terminal half-life (T_1/2_) of 271–272 h in both groups, directly mirroring the overlapping long-term PD protection observed in the functional respiratory assay.

Following SC administration, CSX-1004 achieved an absolute bioavailability (F%) of 92.0%, calculated as the ratio of the dose-normalized ${AUC}_{0-\infty }$ values for the two routes. ${AUC}_{0-\infty }$ was adopted as the basis for this calculation throughout, in preference to the AUC to the last measured concentration, and there are enough concentration-time data available at the elimination phase to capture the elimination half-life to extrapolate ${AUC}_{672-\infty }$ . Also, the terminal phase of an IgG antibody contributes materially to total exposure and is captured only by the extrapolated integral; the corresponding ratio calculated from AUC_0-672_ h is 0.87. The extrapolated portion of ${AUC}_{0-\infty }$ accounted for 16.6% of total exposure after IV administration and 20.9% after SC administration, and estimates of F are therefore interpreted alongside the terminal half-life, which was closely similar between routes (271–272 h) and supports the comparability of the two extrapolations.

### 3.4 Pharmacokinetic model

The final PopPK analysis included a total of 158 serum concentration-time observations collected from eight squirrel monkeys across three treatment conditions (10 mg/kg IV, 40 mg/kg IV, and 40 mg/kg SC), with successive administrations in any individual animal separated by a washout interval of at least 62 days (range 62–128 days), corresponding to more than five terminal half-lives. Systemic disposition of CSX-1004 was best described by a two-compartment open structural model featuring first-order elimination from the central compartment. For the SC administration, a first-order absorption pathway characterized by an absorption rate constant (k_a_) and an absolute bioavailability fraction (F) was successfully incorporated. The residual unexplained variability was optimally captured via a proportional error structure.

#### 3.4.1 Covariate analysis

Body weight and treatment groups were tested as covariates. The treatment group (10 mg/kg IV, 40 mg/kg IV, and 40 mg/kg SC) was identified as the only statistically significant covariate and was included in the final PK model. The parameter-covariate relationships are shown in Formula 3, where $V_{1,i}$ means the predicted central volume of distribution for the *i*th individual animal; $V_{1\_pop}$ represents the population typical volume of distribution for the baseline reference group (10 mg/kg IV); $\beta_{IV40}$ and $\beta_{SC40}$ are covariate fixed effects; $X_{IV40}$ and $X_{SC40}$ serve as an indicator with 0 or one determined by the individual’s group assignment; $\eta_{V1,i}$ is expressed as a random effect. The final model parameters were summarized in Table 2, showing that all PK parameter estimates were with good precision.

$$V_{1,i}=V_{1\_pop}\cdot e^{\left(\beta_{IV40}\cdot X_{IV40}+\beta_{SC40}\cdot X_{SC40}\right)}\cdot e^{\eta_{V1,i}}$$ (3)

**TABLE 2:** Estimates of population pharmacokinetic parameters.

Population parameter (Unit)	Value	RSE (%)	Median of bootstrap^a^ (95% CI)
Fixed effects			
CL (mL/h)	0.16	9.5	0.16 (0.12–0.19)
V_1_ (mL)	19.1	16.7	18.7 (12.9–31.2)
V_2_ (mL/h)	34.8	17.1	35.5 (25.4–55.2)
Q (L/h)	0.78	17.4	0.78 (0.55–1.39)
k_a_ (h^−1^)	0.076	30.2	0.056 (0.015–0.095)
F	0.99	49.3	0.85 (0.61–1.0)
Covariate effects			
βV_1 IV40_	0.68	34.2	0.671 (0.10–1.20)
βV_1 SC40_	0.86	35.6	0.309 (−1.44–1.15)
IIV^b^			
ωCL	0.37 (38.5%)	19.0	0.35 (0.19–0.46)
ωV_1_	0.40 (41.4%)	20.4	0.37 (0.09–0.56)
ωV_2_	0.53 (57.1%)	29.6	0.48 (0.07–0.97)
Residual error			
Proportional error	0.28	7.2	0.27 (0.20–0.33)

Abbreviations: CL, clearance; CV, coefficient of variation; IIV, inter-individual variability; V_1_, volume of distribution in central compartment; V_2_, volume of distribution in the peripheral compartment; Q, intercompartmental clearance, F, fraction absorbed; K_a_, absorption rate constant; ^a^ Based on 1000 bootstrap resampling; ka and F were estimated with RSE, above 30% and their bootstrap distributions are correspondingly wide, so F is reported and interpreted as the bootstrap median with its 95% CI (0.85; 0.61–1.00) rather than as the SAEM, point estimate of 0.99; ^b^ IIV, are presented as mean (CV).

The disposition parameters (CL, V1, V2, and Q), the inter-individual variance terms and the residual error were estimated with good precision, defined *a priori* as a relative standard error (RSE) of 30% or less. The absorption-related parameters were estimated with lower precision (k_a_, RSE 30.2%; F, RSE 49.3%), reflecting the small number of animals contributing SC data and the sparse sampling during the absorption phase; these two parameters are therefore interpreted with corresponding caution, and F is reported below as the bootstrap median with its 95% confidence interval rather than as a point estimate.

#### 3.4.2 Model evaluation

The final PopPK model for CSX-1004 demonstrated robust performance based on goodness-of-fit (GOF) diagnostics, visual predictive checks (VPCs), and bootstrapping (Table 2). GOF plots revealed an absence of systematic bias, with observed concentrations clustering tightly along the line of identity (Figures 3a,b) and individual weighted residuals (IWRES) randomly distributed between −2 and +2 (Figures 3c,d).

**FIGURE 3:**
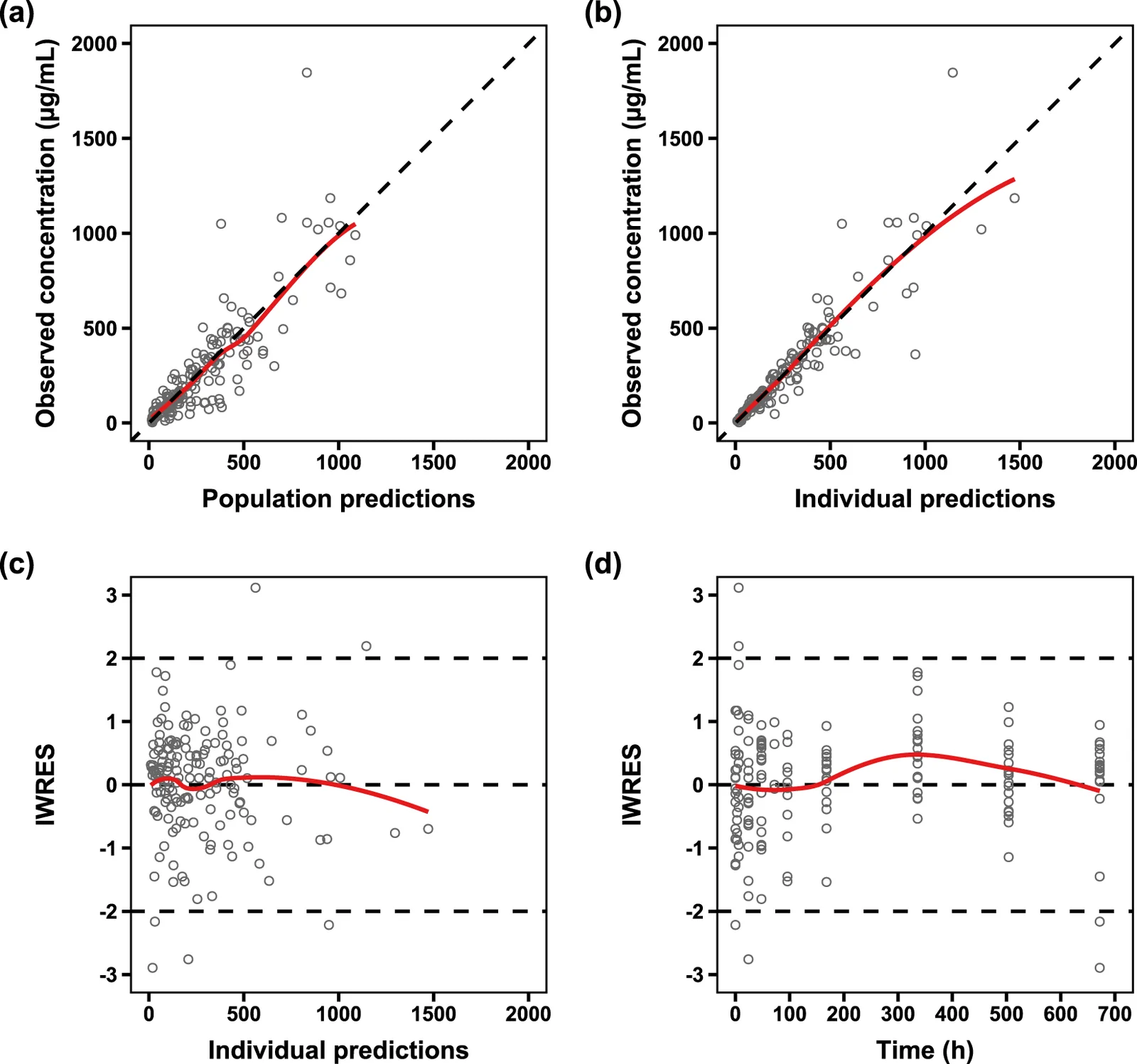
Goodness-of-fit plots of the final population pharmacokinetic model for CSX-1004. **(a)** population predictions versus observations; **(b)** individual predictions versus observations; **(c)** individual predictions versus individual-weighted residuals (IWRES); **(d)** time against IWRES.

VPCs utilizing 500 simulations verified the model’s predictive validity across both linear and semi-logarithmic scales (Figure 4), showing that the empirical fifth, 50th, and 95th percentiles tracked cleanly within their respective 95% simulation-based confidence intervals. Furthermore, model stability was confirmed by a 1,000 bootstrap analysis, where the final SAEM parameter estimates were highly consistent with the bootstrap median values, and all estimated fixed and random effects fell within the 95% confidence intervals.

**FIGURE 4:**
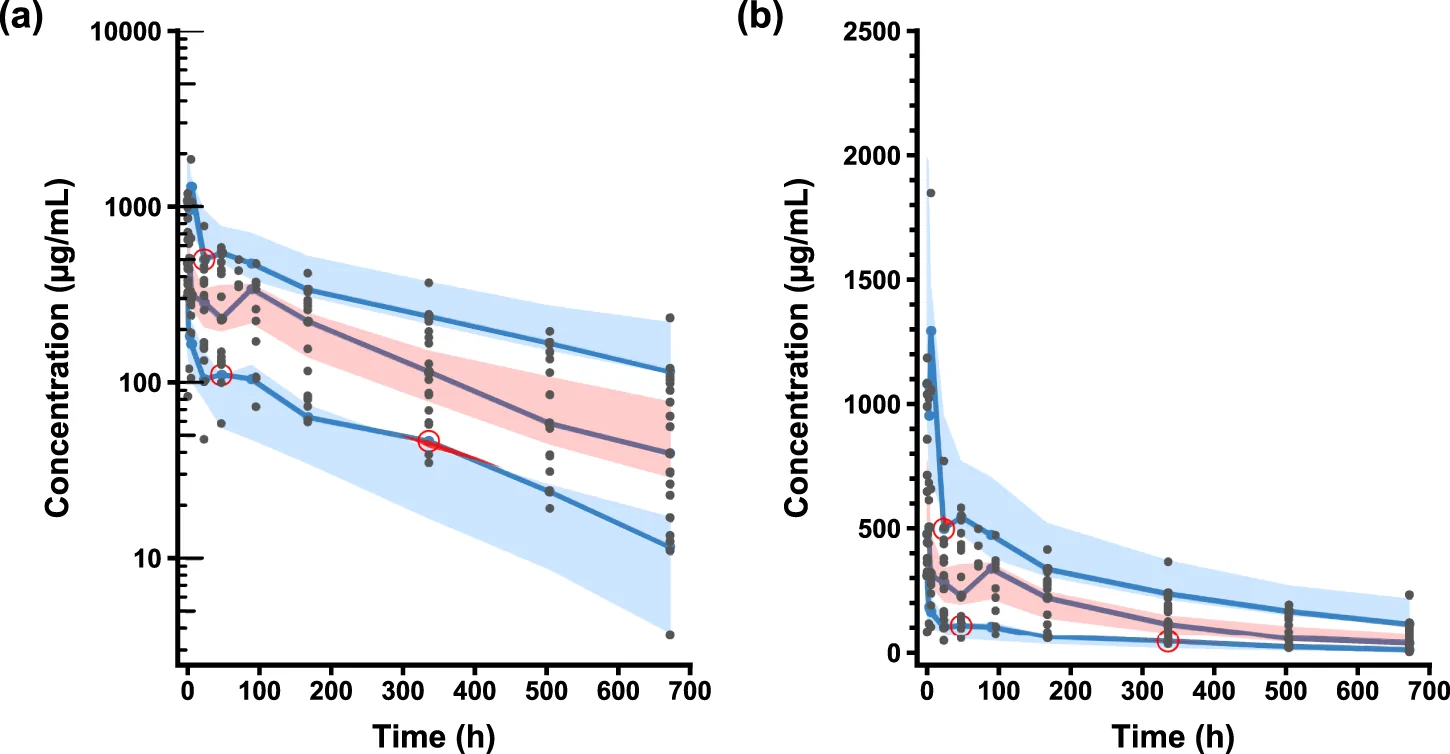
Semi-log scale **(a)** and linear scale **(b)** of visual predictive checks (VPCs) (500 simulations) of the final model for CSX-1004. Dots depict the observed concentrations of CSX-1004. Solid blue lines indicate 95th, 50th, and fifth percentiles of the observed concentrations. Blue shaded regions indicate 95% confidence intervals for the predicted 5th and 95th percentiles, whereas pink shaded regions indicate 95% confidence intervals for the predicted 50th percentiles. Red shaded regions indicate the outlined areas.

### 3.5 Simulations

Population simulations across the 10, 40, and 80 mg/kg dose levels indicated that SC and IV administration produce equivalent systemic exposure beyond the absorption phase (Figure 5). Simulated concentrations differed substantially between routes during the first day: immediately after dosing the median simulated SC concentration was well below the corresponding IV concentration, and the two profiles did not intersect until approximately 17 h post-dose. This early divergence is a genuine and quantitatively important difference rather than a minor one, and it defines the interval during which SC administration cannot substitute for IV administration. Following the absorption phase, the IV and SC concentration-time trajectories were closely superimposed across the terminal elimination phase. Both routes yielded comparable sustained systemic exposures out to Day 28 (672 h), indicating that SC dosing achieves equivalent long-term PK coverage without a meaningful loss of systemic availability. At the 10 mg/kg dose level, simulated concentrations by both routes fell below the 96 μg/mL reference concentration well before Day 28, indicating that the sustained coverage demonstrated at 40 mg/kg is dose-dependent and is not maintained at the lower dose (Figure 5).

**FIGURE 5:**
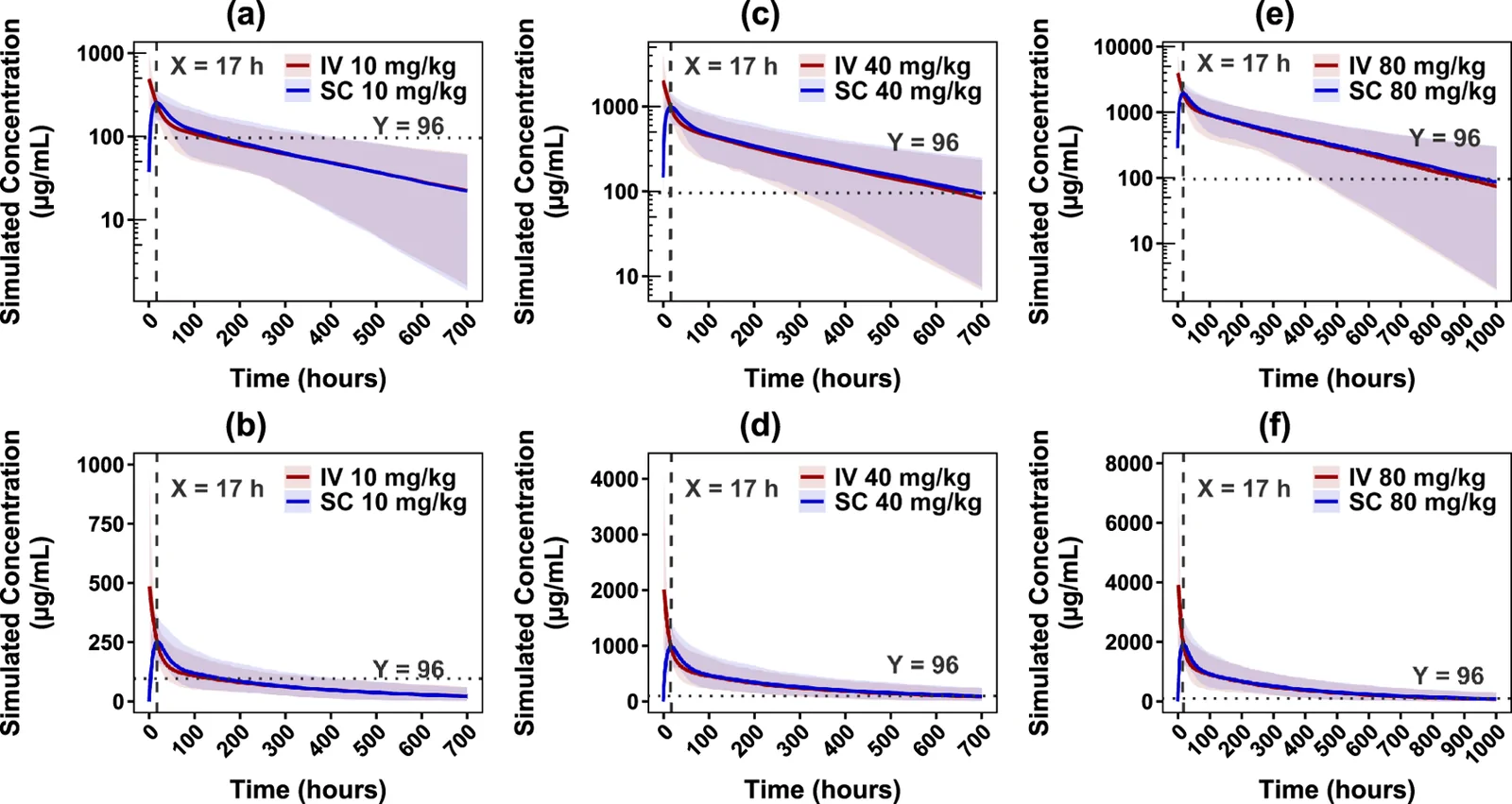
Semi-log scale **(a,c,e)** and linear scale **(b,d,f)** of simulations for 10, 40, and 80 mg/kg following IV and SC administrations based on 500 virtual individuals. The shaded regions indicate the 2.5th to 97.fifth percentiles (95% CI). X = 17 h represents the intersection between IV and SC PK profiles; Y = 96 means the concentration of 96 μg/mL (population estimated concentration at day 28, i.e., 672 h post dose based on IV administration at 40 mg/kg).

## 4 Discussion

Opioid-induced respiratory depression remains the principal cause of mortality associated with fentanyl and other high-potency synthetic opioids in the United States. In the present study, we demonstrate that the CSX-1004 produces robust, long-lasting protection against fentanyl-induced respiratory depression following both IV and SC administration. Using whole-body plethysmography under a hypercapnic 5% CO_2_-air challenge, we observed substantial attenuation of fentanyl’s respiratory suppressive effects that persisted for up to 28 days after a single antibody dose.

Prior to antibody treatment, fentanyl produced the expected dose-dependent decrease in saline minute volume, confirming the sensitivity of the assay to opioid-induced respiratory depression. Following administration of CSX-1004, fentanyl dose-response curves shifted markedly to the right and became substantially flattened, indicating a major reduction in fentanyl potency. The observed >10-fold rightward shift suggests that CSX-1004 effectively sequesters fentanyl in the peripheral circulation, thereby limiting fentanyl penetration into the central nervous system and reducing activation of brainstem opioid receptors involved in respiratory control.

The strongest blockade effects were observed during the first week after antibody administration, consistent with peak systemic antibody concentrations during this period. Although some decline in efficacy occurred over time, significant protection remained detectable through Day 28, supporting the long duration of action of CSX-1004. This prolonged activity is particularly important for overdose prevention strategies, as it may provide extended protection against repeated fentanyl exposure without requiring frequent re-dosing.

A key finding of this study is that SC administration achieved efficacy comparable to IV administration after the initial dosing period. While IV administration produced slightly greater protection at Day 0, due to immediate systemic exposure, SC dosing rapidly achieved equivalent blockade by Day 2 and maintained comparable efficacy throughout the remainder of the study. These findings have important translational implications, as SC administration is clinically more practical, less invasive, and better suited to outpatient and community settings than IV delivery.

The relationship between the PK and PD observations merits explicit consideration, because the two routes differ substantially in serum concentration over the first 24–48 h while differing only modestly in respiratory protection over the same interval. Protection by a neutralizing antibody is governed not by the absolute serum antibody concentration but by the molar ratio of available antigen-binding sites to the quantity of unbound fentanyl entering the circulation. A 40 mg/kg dose of CSX-1004 (molecular weight approximately 150 kDa) corresponds to approximately 267 nmol/kg of antibody, or approximately 533 nmol/kg of antigen-binding sites, whereas the largest cumulative fentanyl dose administered during a challenge session (0.018 mg/kg; molecular weight 336.5 g/mol) corresponds to approximately 54 nmol/kg. Binding-site capacity therefore exceeds the fentanyl molar load by approximately ten-fold at full systemic availability. Estimated from the serum concentrations measured on Day 0, the excess is approximately ten-fold after IV administration and approximately four-fold after SC administration. Because the antibody remains in molar excess by both routes, and because its affinity for fentanyl is picomolar (Bremer et al., 2023), the exposure-response relationship is expected to lie on the shallow, near-saturated portion of its curve, where a several-fold difference in antibody concentration translates into only a small difference in the fraction of fentanyl sequestered. This expectation is consistent with what was observed: the early difference between routes was accurate but modest, and it was confined to the highest fentanyl dose on Day 0, which is precisely the condition under which the molar reserve is smallest and the assay is therefore most sensitive to a difference in antibody concentration. A further consideration is that the ventilatory readout is bounded above by the within-subject saline baseline, so that once sequestration is sufficient to restore ventilation to that baseline no further increase in antibody concentration can produce a measurable increase in the measured response.

It should be stated explicitly that no formal PD or sequential PK/PD model was developed in this study, and the present findings are therefore not described as establishing a PK/PD correlation. What the data support is an exposure-response consistency: the interval over which serum concentrations converged between routes coincides with the interval over which the respiratory dose-effect functions converged, and serum concentrations remained above the concentration associated with sustained protection through Day 28 by both routes. A formal PK/PD model was not attempted because the PD design provides observations on six occasions at a single antibody dose level in a small number of animals, with the response saturated over most of that range, which is insufficient to identify the parameters of an E_max_ or receptor-occupancy model with useful precision. Formal characterization of the exposure-response relationship will require a study in which several antibody dose levels are examined, so that the PD readout spans the ascending portion of the concentration-effect curve rather than its plateau.

The population model was developed for purposes that non-compartmental analysis cannot serve. Non-compartmental analysis yields a single point estimate of bioavailability with no measure of its uncertainty and no separation of between-subject from residual variability. The population approach provides an estimate of F with an associated confidence interval, partitions inter-individual variability in clearance and volume of distribution, permits formal testing of body weight and treatment group as covariates, and, most importantly for the translational question posed here, allows simulation of concentration-time profiles at dose levels and by routes that were not studied experimentally, together with the probability that a given regimen maintains concentrations above the protective threshold through Day 28. The bioavailability estimate is one output of that framework rather than its rationale.

The pharmacokinetic profile of CSX-1004 following both IV and SC administration demonstrates characteristics typical of IgG mAbs, supporting its potential as a durable therapeutic agent for mitigating the effects of fentanyl exposure. Following IV administration (40 mg/kg), CSX-1004 exhibited observed concentration (C_0_) of 1009.3 ± 102.6 μg/mL at the first time-point after dosing and a terminal half-life of 272.1 ± 92.5 h, with a total systemic exposure ( ${AUC}_{0-\infty }$ ) of 198,819.0 ± 24,894.8 h*µg/mL. The apparent volume of distribution (V_z_ = 78.6 ± 22.1 mL/kg) suggests limited tissue distribution beyond the vascular compartment, consistent with the limited extravascular diffusion observed for IgG-class antibodies. The slow elimination kinetics are likely attributable to FcRn-mediated recycling, which minimizes antibody catabolism and prolongs the terminal half-life (t_1/2_). The extended T_max_ observed after SC dosing, by contrast, is a property of the absorption process rather than of FcRn-mediated recycling and reflects the slow diffusion and lymphatic uptake of a macromolecule from the SC space. Following SC administration, CSX-1004 exhibited gradual systemic absorption with an absorption rate constant (K_a_) of 0.076 h^-1^, reaching a C_max_ of 491.7 ± 50.4 μg/mL at 48.0 ± 19.6 h (T_max_), post-dose. Importantly, the sustained plasma concentrations achieved after SC administration indicate efficient systemic absorption and support the potential for less frequent dosing in chronic or prophylactic treatment settings.

The total systemic exposure ( ${AUC}_{0-\infty }$ ) was 198,819.0 ± 24,894.8 h μg/mL for the IV route and 182,993.7 ± 52,563.7 h μg/mL for the SC route, demonstrating robust systemic exposure following both modes of administration. The absolute bioavailability of CSX-1004 after SC dosing was 92.0% on this basis, indicating efficient absorption with modest systemic loss. This high bioavailability suggests that SC administration may be clinically preferable to IV delivery in scenarios emphasizing ease of use and patient compliance. The higher T_max_ observed after SC administration may be influenced by distribution kinetics or sampling intervals and is within the typical 2–7-day T_max_ (48.0 ± 19.6 h) of SC IgG mAbs. The loss of approximately 8% associated with SC dosing is consistent with the presystemic catabolism and incomplete lymphatic recovery typically reported for subcutaneously administered IgG.

It is useful to establish a long-acting antibody relative to naloxone rather than in opposition to it. Naloxone is a competitive MOR antagonist with rapid onset and a plasma half-life of approximately 1–2 h; these properties make it the appropriate agent for reversing an overdose already in progress, but they also mean that its protective effect dissipates well before that of ultrapotent or long-acting synthetic opioids, so that renarcotization and repeated dosing are recognized clinical problems (Dahan et al., 2024; Moss and Carlo, 2019; Rzasa Lynn and Galinkin, 2018). CSX-1004 operates by a different mechanism and on a different time scale. It sequesters fentanyl in the peripheral circulation before receptor engagement rather than displacing it afterward, it does not act at the opioid receptor and therefore would not be expected to precipitate withdrawal in opioid-dependent individuals, and a single administration confers protection for weeks rather than for approximately an hour. The two approaches are therefore complementary rather than competing. Naloxone remains the intervention for acute reversal; a long-acting mAb is directed at the interval between exposures, during which the risk of a subsequent fatal exposure is high and no pharmacological protection is currently available. The SC route evaluated here is relevant precisely to that prophylactic role, since it can be administered in outpatient, community, or post-incarceration settings that lack venous access, and its slow absorption is of little consequence when protection is established in advance of exposure rather than in response to it.

Several limitations should be acknowledged. The PK dataset comprises 158 observations from eight squirrel monkeys, supplemented with historical IV data from a previous study in the same species and subjects (Bremer et al., 2023). A dataset of this size materially constrains the statistical power to detect covariate effects. Body weight, which is a physiologically expected determinant of antibody clearance and volume of distribution, was not identified as a significant covariate, and its absence from the final model should be interpreted as a limitation of the available information rather than as evidence that body weight does not influence CSX-1004 disposition; the narrow body-weight range of the study animals (0.65–1.0 kg) further limits the ability to resolve such an effect. The absorption-related parameters were correspondingly imprecise (k_a_, RSE 30.2%; F, RSE 49.3%), and the bootstrap distribution of F was wide (median 0.85; 95% CI 0.61–1.00); SC bioavailability is therefore not described as approaching 100%. The extrapolated portion of ${AUC}_{0-\infty }$ exceeded 20% for the SC route, which introduces a further source of uncertainty into the non-compartmental estimate of F. The PD component was conducted at a single antibody dose level, which precludes formal exposure-response modeling, and the use of historical IV PK data introduces the possibility of between-study differences in assay performance even though the same cohort of subjects were used. Finally, protection was assessed against fentanyl alone. Cross-reactivity with fentanyl analogs was not evaluated in the present study. Although previous work established that CSX- 1004 binds with picomolar affinity to sixteen fentanyl analogs with no appreciable cross-reactivity with morphinan opioids, the MOR antagonists, or endogenous opioid peptides. Furthermore CSX-1004 was shown to reverse carfentanil-induced respiratory depression in mice (Bremer et al., 2023). Testing each analog *in vivo* is not feasible, but the breadth of that binding profile makes it reasonable to expect that the protection demonstrated here against fentanyl would extend to structurally related analogs.

Collectively, these PK findings demonstrate that CSX-1004 possesses favorable *in vivo* characteristics, including high systemic availability, sustained plasma exposure, and suitability for both IV and SC administration routes.

## 5 Conclusion

CSX-1004 significantly attenuated the respiratory depressant effects of fentanyl, producing a marked rightward shift in the fentanyl dose-response curve that persisted for up to 28 days after a single administration. The greatest blockade effect was observed during the first 7 days, with a gradual reduction over time while maintaining partial protection through Day 28.

Importantly, SC administration produced efficacy comparable to IV administration after the initial dosing period. Although IV dosing showed slightly greater protection at Day 0, likely due to faster systemic availability, SC administration achieved similar blockade from Day 2 onward. These findings suggest that SC delivery may represent a practical and clinically feasible route for long-acting protection against fentanyl-induced respiratory depression.

A successfully developed population PK model indicated that SC and IV administration of CSX-1004 produce equivalent systemic exposures once the absorption phase is complete. Absolute bioavailability after SC dosing was high but was not established as complete: non-compartmental analysis based on ${AUC}_{0-\infty }$ gave 92.0%, and the population estimate was imprecise (typical value 0.99, RSE 49.3%; bootstrap median 0.85, 95% CI 0.61–1.00). Moreover, CSX-1004 exhibited dose-dependent efficacy in preventing fentanyl respiratory depressant effects based on our previous study, with comparable therapeutic protection sustained through Day 28 across the 40 mg/kg IV and SC groups. The alignment between the interval over which systemic exposures converged and the interval over which respiratory protection converged is consistent with an exposure-driven effect; however, no formal PK/PD model was developed, and these data should therefore be understood as demonstrating exposure-response consistency rather than as establishing a quantitative PK/PD relationship. Subject to the absorption lag over the first 24–48 h, the SC route is a practical alternative to IV administration for long-acting prophylaxis.

Overall, the prolonged duration of action, the magnitude of the respiratory protective effect, and the feasibility of SC dosing support the therapeutic potential of CSX-1004 as an immunotherapy directed principally at the prevention, rather than the acute reversal, of fentanyl overdose.

## Data Availability

The raw data supporting the conclusions of this article is available at https://doi.org/10.6084/m9.figshare.31069939.
